# Synthesis of descriptive sensory attributes and hedonic rankings of dried persimmon (*Diospyros kaki* sp.)

**DOI:** 10.1002/fsn3.537

**Published:** 2017-11-09

**Authors:** Rebecca R. Milczarek, Rachelle D. Woods, Sean I. LaFond, Andrew P. Breksa, John E. Preece, Jenny L. Smith, Ivana Sedej, Carl W. Olsen, Ana M. Vilches

**Affiliations:** ^1^ Healthy Processed Foods Research Unit Western Regional Research Center United States Department of Agriculture – Agricultural Research Service Albany CA USA; ^2^ Department of Food Science & Technology University of California, Davis Davis CA USA; ^3^ National Clonal Germplasm Repository United States Department of Agriculture – Agricultural Research Service Davis CA USA

**Keywords:** Consumer, drying, persimmon (*Diospyros kaki*), sensory evaluation, value‐added product

## Abstract

This work aimed to characterize the sensory attributes of hot air‐dried persimmon (*Diospyros kaki*) chips, correlate these attributes with consumer hedonic information, and, by doing so, present recommendations for cultivars that are most suitable for hot‐air drying. A trained sensory panel evaluated dried persimmon samples (representing 40 cultivars) for flavor, taste/aftertaste, and texture. In addition, in each of two tests conducted in different years, more than 100 consumers provided hedonic evaluations of 21 unique samples in a ranking task with a balanced incomplete block design. A partial least squares regression model correlating the mean hedonic ranking to the trained panel data was developed using the data from the first consumer panel. The predictions from the model were correlated with the second panel to verify the model. It was found that including taste, aftertaste, and texture data (but not specific flavor attribute data) produced a predictive model (Spearman's ρ=0.83). This indicates that flavor is likely secondary to taste and texture in dried persimmon chips. Using the validated predictive model, 6 of the 40 persimmon cultivars tested are recommended for a dried chip product; these cultivars are ‘Fuyu’, ‘Lycopersicon’, ‘Maekawa Jiro’, ‘Nishimura Wase’, ‘Tishihtzu’, and ‘Yotsumizo’.

## 1 INTRODUCTION

Persimmon (*Diospyros kaki*) is a subtropical tree fruit with worldwide commercial production of 4.6 million metric tons in 2013 (UN FAOSTAT, 2016). In the United States, California accounts for almost all persimmon production (Kader & Arpaia, 2002). Over 100 cultivars of persimmon have been identified (Sugiura, Tao, & Tomana, 1988), and, based on their astringency at harvest and pollination state, these cultivars can be separated into three categories: “astringent,” “nonastringent,” and “pollination variant.” All three categories of persimmon can first be harvested when commercial ripe—that is, when the fruit is firm and the skin has changed color from green to yellowish‐green, yellow, orange, or reddish‐orange (cultivar dependent) (Crisosto, 1999). Nonastringent cultivars are palatable immediately upon harvest; a common nonastringent persimmon cultivar grown in the United States is ‘Fuyu’. If the fruit of pollination variant (referred to as simply “variant” hereafter^1^ ) cultivars are pollinated in the spring (and thus have seeds upon maturation), they will behave like nonastringent cultivars and be palatable immediately upon harvest. In contrast, astringent and nonpollinated variant cultivars must undergo a further deastringency process known as “mellowing” or “bletting” in order to be palatable in raw form. A common astringent persimmon cultivar grown in the United States is ‘Hachiya’.

Several options are available for the deastringency process, including simple softening at room temperature, freeze/thaw treatment, ethanol treatment, and controlled atmosphere treatment with carbon dioxide or nitrogen (Arnal, Besada, Navarro, & Salvador, 2008; Besada & Salvador, 2011; Pesis, Levi, & Ben‐Arie, 1986; Taira, Ono, & Otsuki, 1998). From a sensory standpoint, the flavor attributes of fresh ‘Fuyu’ (Lyon et al. 1992) persimmons have been reported, and several studies report the sensory‐evaluated astringency of fresh persimmons before and after deastringency treatments (Arnal et al., 2008; Novillo, Besada, Gil, & Salvador, 2013; Novillo, Gil, Besada, & Salvador, 2014; Taira, Ono, & Matsumoto, 1997; Taira et al., 1998).

In addition to their consumption in fresh form, some astringent persimmon cultivars are amenable to drying into “hoshi‐gaki”—a confectionary delicacy with its origins in East Asia. Hoshi‐gaki are prepared by tying ripe astringent persimmons on a string and allowing them to dry outdoors for 2–4 weeks; hand kneading of the drying fruit is sometimes performed to facilitate even moisture distribution. The resulting product (30%–50% moisture content) has a texture similar to that of jelly candy and a naturally‐occurring powdery sugar coating; the astringency is also completely removed by the drying process (Sugiura & Taira, 2009).

Persimmons are a rich source of Vitamin C, carotenoids, and polyphenolic compounds. In vivo and in vitro studies of these dietary components suggest a relevant role of this fruit in protection against free radicals and prevention of some human diseases (Giordani, Doumett, Nin, & del Bubba, 2011). The overall aim of this study is to encourage more consumption of persimmons. Developing a dried chip‐style product provides persimmon growers an option for preserving and marketing their fruit without using the (labor‐ and time‐intensive) hoshi‐gaki process. Dried apple chips are an analogous product that has seen widespread distribution, and the sensory properties of this product have been well‐characterized (Konopacka & Plocharski, 2007; Sham, Scaman, & Durance, 2001; Velickova, Winkelhausen, & Kuzmanova, 2014).

Exploratory studies of hot air‐dried and sun‐dried sliced persimmon products have been conducted by a number of groups (Cárcel, García‐Pérez, Sanjuán, & Mulet, 2010; Igual, Castelló, Roda, & Ortolá, 2011; Karakasova, Babanovska‐Milenkovska, Lazov, & Stojanova, 2013; Park et al., 2006; Senica, Veberic, Grabnar, Stampar, & Jakopic, 2016). However, of the more than 100 persimmon cultivars that exist, these studies have collectively involved only a small subset: ‘Triumph’ (Park et al., 2006), ‘Rojo Brillante’ (Cárcel et al., 2010; Igual et al., 2011), ‘Tipo’ (Senica et al., 2016), and an unspecified astringent cultivar (Karakasova et al., 2013). These studies included chemical analysis of the fruit, performed before and after drying. However, evaluation of the dried products by consumers was not reported. Also, each of these studies involved persimmons collected at a single point during the harvest season; it is possible that the quality of the dried products would have been different for early‐ and late‐harvest source fruit.

Thus, the purpose of the present work was to assess the suitability of 40 cultivars of persimmon (harvested at multiple time points and from multiple sources, when possible) for hot‐air drying into a chip‐style product. Assessments of the taste/aftertaste, flavor, and texture of the dried products were obtained from a trained sensory panel, and these results were correlated with the hedonic rankings of the products by 150 consumers in each of 2 years. The challenges of the large sample set and the timing of the consumer panels in the middle of the harvest season were addressed by the methods of a balanced incomplete block design and predictive partial least squares regression model, respectively.

## 2 MATERIALS AND METHODS

### 2.1 Persimmon samples

Fifty‐four fresh persimmon samples, consisting of ~200 fruit each, were harvested in Fall 2015 and dried for this study (Table 1). The persimmon samples included 40 distinct cultivars: 11 astringent, 13 nonastringent, and 16 variant. The persimmon samples were acquired from four California sources: the United States Department of Agriculture – Agricultural Research Service National Clonal Germplasm Repository for Fruit & Nut Crops (USDA‐ARS NCGR, Davis, Calif., U.S.A.), Commercial Source #1 (L.E. Cooke, Co., Visalia, CA, USA), Commercial Source #2 (Oak Acre Farms, Live Oak, CA, USA), and Commercial Source #3 (Mr. O. Bertolero, Santa Rosa, CA, USA). These sources are denoted as R, C‐1, C‐2, C‐3 in Table 1. For additional detail, the accession numbers for the NCGR samples and California counties of origin for the commercial samples are listed in Supplemental Table S1). For some cultivars and sources, there was enough fruit available to collect multiple samples throughout the season; in these cases, the sample harvests were spaced apart by a minimum of 12 days. Persimmons were hand‐harvested when commercial ripe—that is, when the exterior color had changed from green to yellowish‐green, yellow, orange, or reddish‐orange (cultivar dependent). Persimmons were packed directly into boxes with plastic liners that separated and cushioned each fruit. Within 24 hr of harvest, the boxes of fruit were transported to the USDA‐ARS laboratory in Albany, CA, USA via pickup truck (Source R) or overnight commercial shipping (Sources C‐1, C‐2, and C‐3).

**Table 1:** Sources, astringency types, number of harvests, and consumer evaluation status of the persimmon cultivars in this study

Cultivar	November 2015 consumer panel (CT1) evaluated dried form	November 2016 consumer panel (CT2) evaluated dried form	Number of harvests	Astringency type	Source
[unnamed]	+		1	A	R
Akoumanzaki		+	1	V	R
Brazzale^a^			1	V	R
Chienting	+		2	V	R
Chocolate	+		1	V	C‐1
Emon	+		1	V	R
F‐444	+		2	N	R
Fennio		+	1	A	R
Fujiwaragosho	+	+ (1st, 2nd)	2	V	R
Fuyu	+		2	N	R
Fuyu		+	1	N	C‐2
Fuyu	+		3	N	C‐3
Fuyu Imoto		+	1	N	C‐1
Fuyu Jiro		+	1	N	C‐1
Giant Fuyu		+	1	N	C‐1
Gofu		+	1	V	R
Great Wall	+		1	A	R
Hachiya		+	1	A	C‐1
Hanagosho		+	2	N	R
Ichidagaki	+		1	A	R
Ichikeijiko	+	+ (1st, 2nd)	2	A	R
Izu	+		2	N	R
Izu	+		1	N	C‐1
Jiro	+		1	N	R
Korean	+		2	A	R
Lampadina	+		1	V	R
Lycopersicon	+		1	A	R
Maekawa Jiro	+		1	N	R
Mandarino		+	1	V	R
Maru		+	1	N	C‐1
Mishirasu	+	+ (1st, 2nd, 3rd)	3	A	R
Moro		+	1	V	R
Muraya	+		2	V	R
Nishimura Wase	+	+	1	V	C‐1
Okugosho		+	1	V	R
Sangokuichi	+		1	V	R
Suruga		+	1	N	R
Syouro	+		1	V	R
Tamkam		+	2	N	R
Tishihtzu		+	1	A	R
Vainiglia		+	1	V	R
Yeddo	+		1	N^b^	R
Yotsumizo	+		1	A	R

“+” for the two consumer panel columns indicates that the sample was included in the indicated consumer hedonic ranking portion of the study. (All samples were evaluated by the trained sensory panel). In those same columns, “+” with no modifier indicates that the first harvest of the cultivar was used; the modifiers “1st,” “2nd,” and “3rd” indicate cases where multiple harvests were used. Astringency Type: A, astringent; N, nonastringent; V, variant. Source: R, research plot (National Clonal Germplasm Repository, Davis, CA, USA); C‐X, commercial source X.

a There was not a sufficient amount of cultivar ‘Brazzale’ available for either consumer panel. However, this cultivar was evaluated by the trained panel.

b Evidence from this study suggested that cultivar ‘Yeddo’ is a nonastringent cultivar; this differs from the classification of “variant” given by other publications (Camp & Mowry, 1929; Ryerson, 1927).

### 2.2 Drying method

**Figure 1:**
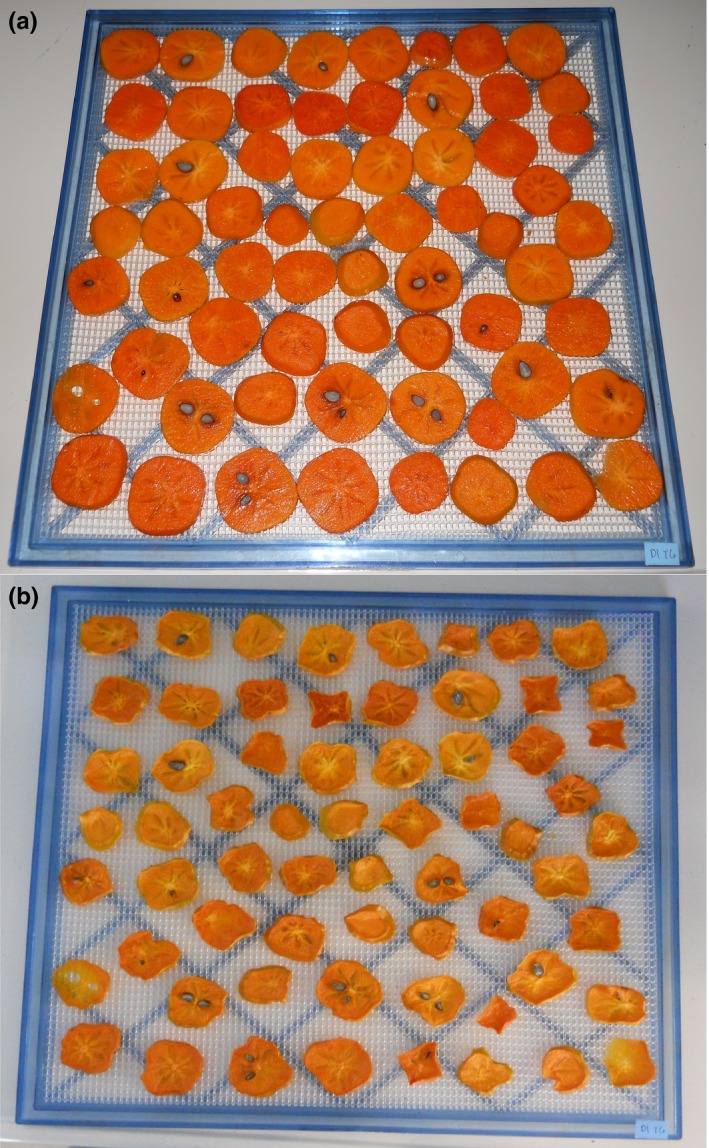
Persimmon sample (“Yotsumizo”, harvest date: 8 October 2015, source: National Clonal Germplasm Repository, Davis, CA, USA) before (a) and after (b) hot‐air drying

Upon receipt, the persimmon samples were hand‐sorted to remove visibly damaged fruit and then stored for an average of 8 days. Following best practices for this commodity (Crisosto, 1999), nonastringent cultivars were stored in an incubator set at 18°C, and astringent and variant cultivars were stored in a refrigerator set at 2°C. On the day of processing, persimmons were washed in tap water to remove surface soil and then sanitized in a 200 ppm chlorine solution. Slices of 5 mm thickness were cut with a commercial meat slicer (Model 1612P, Hobart, Troy, OH, USA). If present, seeds and seed fragments were left in the slices, since practice runs revealed that the seeds were easier to remove after drying than before drying. The slices were arranged in a single layer on the trays of a commercial dehydrator (Model 2924T, Excalibur Dehydrator, Sacramento, CA, USA) and dried at 52°C (125°F) for 18 hr. The dried slices were stored at ambient temperature in sealed metallized polyester film pouches. Before‐ and after‐drying photos of a typical persimmon sample are shown in Figure 1.

### 2.3 Trained panel sensory evaluation

Descriptive sensory analysis was conducted on the 54 samples of dried persimmons in February through March 2016 (i.e., after the entire persimmon harvest season was complete and all samples were dried). Due to the lengthy time commitment and limited panelist availability, eight adult panelists (six female, two male, aged 35–65) were recruited from the USDA‐ARS laboratory (Albany, CA, USA), rather than the customary 10–12. Most had no previous sensory evaluation experience and were selected based on their availability and willingness to participate in the evaluation of dried persimmons over the 8‐week course of the study and their demonstrated basic sensory acuity. All panelists participated in at least four, 1‐hr training sessions, which covered 8 texture attributes, 4 taste attributes (sweet, sour, salty, bitter), aftertaste (astringency), overall flavor intensity, and 21 individual flavor attributes, listed in Supplementary Table S2. The attributes used for texture and taste were determined from the literature (Bourne, 2002; King et al., 2012); the attributes for flavor and astringency were benchtop tested and experimentally determined. Definitions for the texture attributes can be found in Table S3. Panelists were considered adequately trained when they could correctly identify all 21 flavor attributes on the first try and correctly identify and rank 4 different concentrations of each of the five taste and aftertaste attributes. Additionally, the panelists practiced the testing procedure and use of the scales twice prior to evaluating the persimmon samples. The panelists received no compensation other than snacks at the end of each session.

Evaluation of the dried persimmon samples took place in isolated booths. The panelists were given one whole slice plus one “wedge” (1/4 to 1/3 of a slice) to assess the eight texture attributes. They were then presented with an additional half slice with which to evaluate the individual flavor attributes, taste, aftertaste, and overall flavor intensity. The panelists were instructed to taste the skin and flesh of each sample and expectorate all samples. Panelists received 12 products per session, constituting four samples evaluated in triplicate. Products were randomized and presented in black soufflé cups labeled with three‐digit random codes. The panelists were instructed to cleanse their palates between samples with filtered water and unsalted water crackers (Carr's, Carlisle, UK)—a palate cleansing approach recommended for high‐astringency foods like wine (Ross, Hinken, & Weller, 2007). To reduce fatigue, no more than 12 samples were evaluated in any given session, and all sessions lasted a maximum of 1 hr.

The texture attributes were rated on a 15 cm unstructured line scale with specific product anchors throughout the scale. The taste, astringency, and overall flavor intensity attributes were also rated on a 15 cm unstructured line scale, but using only “low” and “high” at either end as anchors. Individual flavor attributes were evaluated using “check‐all‐that‐apply” (CATA), which has been used by several recent studies with trained assessors (Campo, Ballester, Langlois, Dacremont, & Valentin, 2010; King et al., 2012; Lazo, Claret, & Guerrero, 2016). This method was chosen since it was thought that the persimmon flavors would be more readily assessed as “present” or “absent,” versus having the intensity of these attributes indicated on a scale. Although, under this method, an individual panelist marks only the presence or absence of a specific attribute for a specific sample, aggregating repeated CATA assessments across multiple panelists leads to relative intensity information (Campo et al., 2010)—for example, an attribute that is selected 90% of the time is clearly more intense than an attribute that is selected only 5% of the time.

### 2.4 Consumer tests

At the time of the first consumer test (early November 2015), there were only 25 dried persimmon samples available. The remaining samples had not yet been harvested. As it was not feasible for each consumer to evaluate all 25 samples, a balanced incomplete block design was used. This type of design has been used for other food products when it is desirable to reduce the assessment load on consumers (Bower & Whitten, 2000). The balanced incomplete block design used was (*v*=25, *b*=30, *k*=5, *r*=6, λ=1) where *v* is the number of products, *b* is the number of panelists in a block, *k* is the number of products each panelist evaluates, *r* is the number of times each sample appears across all blocks, and λ is the number of times each pair of samples appears across all blocks. For the consumer tasting, five full replicates (150 total panelists) of the balanced incomplete blocked design were prepared, and the presentation order for each block was randomized. The same experiment design and number of samples (25) were also used for the second consumer test, though the composition of the sample set was different (more details are given in later in this section). In addition to hedonic assessment, basic demographic information and data about consumption frequency of fresh persimmons and dried fruit were collected.

Both consumer tests were carried out at the National Clonal Germplasm Repository (Davis, CA, USA) annual tasting of persimmons and pomegranates in early November 2015 (Consumer Test 1—CT1) and early November 2016 (Consumer Test 2—CT2). This annual event is open to the public, and the attendees have an interest in fruit tasting but may or may not regularly consume dried fruit and/or persimmons. For CT1, the consumer group was 58% female/42% male and ranged in age from 10 to 70. In terms of fresh persimmon consumption during the September‐to‐December harvest season, the group was nearly evenly divided among the six frequency categories offered—from “never” (16%) to “daily” (12%). For dried fruit consumption, the majority (57%) of the group consumed dried fruit between 1–3 times/month and 3–5 times/week. The CT2 consumer group had similar distributions of age, gender, and dried fruit consumption to that of the CT1 group. In terms of fresh persimmon consumption frequency, however, the CT2 group had a more concentrated subset in the “1–3 times/month” category (27%) compared to the earlier group.

During the test, each consumer panelist was given a bag containing their five samples and the order in which they were to taste the samples. In each year, 150 sample bags were distributed, but not all score sheets were returned. This limitation meant that—out of 150 possible panelists—data were collected for only 135 and 136 panelists for CT1 and CT2, respectively. In each year, three complete blocks could be constructed from the responses. For a fourth block, 28 and 26 (of the required 30) responses were available for CT1 and CT2, respectively. The missing data points were imputed in order to form a final data set which consisted of responses for four blocks (120 consumers) for each year, with 2 and 4 responses imputed for CT1 and CT2, respectively.

Scoresheets for the consumer test were identical for CT1 and CT2 except for the area in which the consumer ranked his or her five samples. For CT1, this area had a simple ranking task: ordering the five samples from “Like the Least” to “Like the Most.” The consumer was instructed to place preprinted sample‐number stickers in the designated five spaces according to the consumer's preference for the samples. This basic ranking approach was chosen for several reasons. It was known beforehand that the attendees at the event would vary widely in age and product evaluation experience, and ranking can be performed even by consumers who are unfamiliar with product rating scales. In addition, ranking using preprinted stickers helped simplify the data collection and reduced the risk of panelists’ failing to evaluate 1 or more samples out of the set of 5 (a particularly important issue for a balanced incomplete block design). However, this method had inherent limitations. It captured consumer preference information but not necessarily consumer acceptance information, since the task was a forced ranking of five samples, whether all the samples were well‐liked, disliked, or somewhere in between. To capture both preference and liking information, the task was altered for CT2. In the CT2 scoresheet, 15 possible sticker‐placement spaces were distributed evenly along a line anchored with “Dislike” at the far left, “Neither Dislike Nor Like” at the center, and “Like” at the far right. Thus, panelists were still forced to rank the five samples, but a liking rating of 1 (“Dislike”) to 15 (“Like”) was obtained simultaneously. In short, CT1 was comprised solely of a ranking task while CT2 was comprised of a combined ranking/rating task.

For CT1, the 25 samples were, by necessity, the first harvests of 25 cultivars that were available at the time of the test. For CT2, however, all 54 samples were available (and had been evaluated by the trained panel earlier in 2016—a timeline of the study is given as Supplemental Figure S1). So, a subset of 25 samples was chosen for CT2 based on the following criteria:

- cultivars that had not been evaluated by consumers in CT1 (16 samples)
- a sample of cultivar ‘Fuyu’ from a commercial source that had not been evaluated by consumers in CT1 (one sample)
- “anchor” cultivars known to be low‐, medium‐, and high‐preference from the results of CT1 (three samples)
- Second harvests (when available) of the “anchor” cultivars (two samples)
- First, second, and third harvests of cultivar ‘Mishirasu’—a cultivar whose first harvest was low‐preference in CT1 but whose preference at third harvest was predicted to be high (see Section 3.4 for additional details) (three samples)

The samples used in CT1 and CT2 are indicated in Table 1 with “+” symbol.

### 2.5 Statistical analyses

Trained panel sensory data were summarized using multi factor analysis (MFA) and hierarchical clustering on principal components (HCPC). The MFA and HCPC were carried out using the FactoMineR package in R (Husson, Josse, Le, & Mazet, 2015) with the flavor, taste, and texture data being considered different groups of variables. The total count for each flavor attribute for each product was used as the response and was considered frequency data. The mean ratings for the taste and texture data were treated as continuous data and were scaled before analysis. Based on visual examination of the scree plot, the first five components from the MFA were used for the HCPC. The number of clusters for the HCPC was determined using the default method for FactoMineR. By this method, a hierarchical tree is built. The sums of the within‐cluster inertia are then calculated for each partition. The suggested partition is the one with the higher relative loss of inertia (*i* [clusters *n*+1]/*i* [cluster *n*]) (Husson et al., 2015).

The consumer ranking data from CT1 were initially analyzed using Durbin's test with a least significant difference (LSD) test to determine which samples were different at *p*<.05 (Conover, 1999). The trained panel descriptive data and the CT1 hedonic data were correlated using partial least squares regression (PLSR) using the pls package in R (Mevik, Wehrens, & Liland, 2015). The PLSR model correlated the mean rank of the dried persimmon samples to the scaled mean values for all texture and taste data and the scaled frequency for all flavor data except for the “chocolate” and “coconut” attributes, which were removed as they had a frequency count of 0 for the products tested. The model used the first two components of the PLSR; this number of components was chosen to minimize the root mean square error of prediction (RMSEP). In addition, a second PLSR model was constructed using the trained panel attributes *without* the specific flavor attributes; this sparse model used only the first component of the PLSR, again minimizing RMSEP. Both models were applied to both CT1 and CT2 data and validated using leave‐one‐out cross validation. All graphs were constructed using the R package ggplot2 (Wickham, 2016); all other statistics were conducted in R (R Core Team 2015).

## 3 RESULTS AND DISCUSSION

### 3.1 MFA on trained panel sensory data

**Figure 2:**
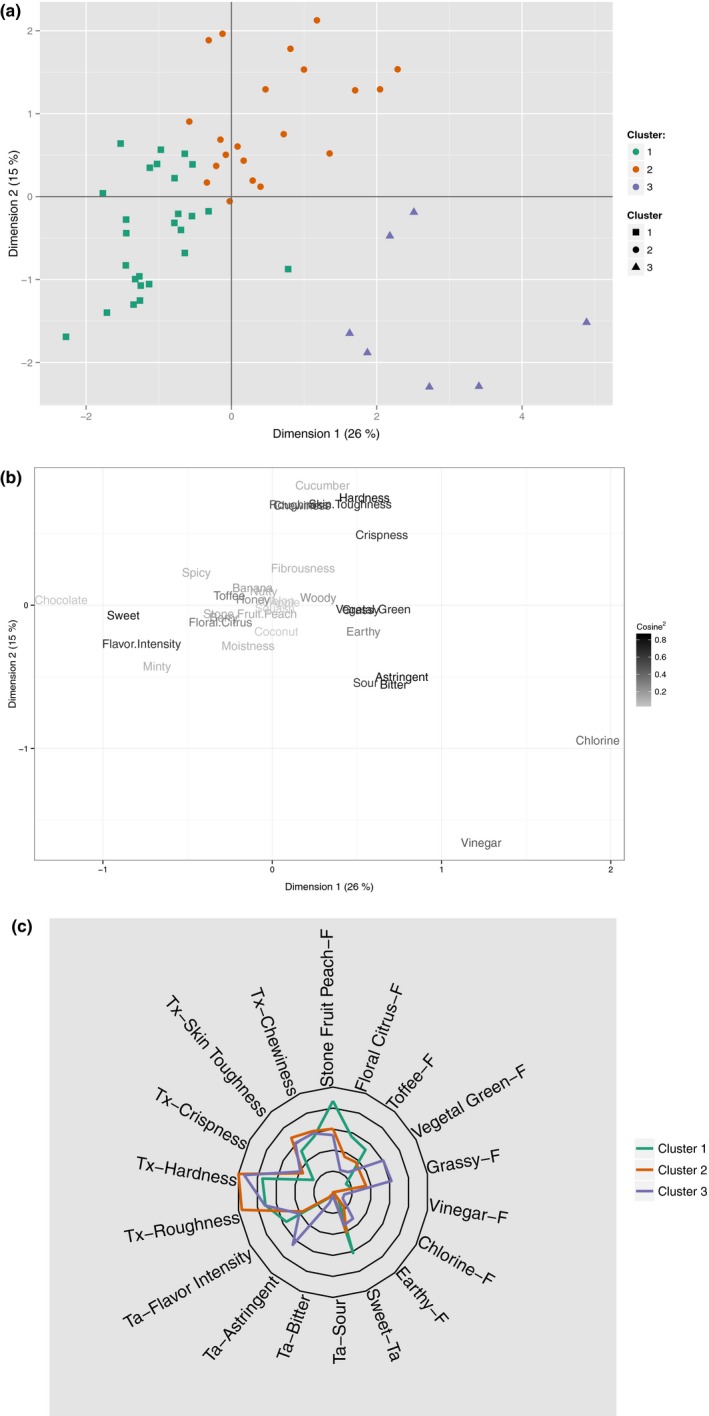
Multi factor analysis [MFA] and hierarchical clustering on principle components [HCPC] results from trained sensory panel. The products (a) and sensory attributes (b) are depicted on the first two MFA dimensions. In (b), darker text is better represented by the first two dimensions of the MFA. Attributes that differ between the clusters (p < 0.05) are shown in a spider plot (c). In (c), prefixes and suffixes F = flavor, Ta = taste/aftertaste, Tx = texture

The MFA with HCPC identified three sensory clusters (Figure 2). Figure 2a shows the products on the first two dimensions of the MFA representing 41% of the total variance in the dataset; sample cluster is denoted by both shape and color of the point. Figure 2b shows the corresponding placement of the sensory attributes for the MFA, with the darkness of the text of the attribute representing how well the biplot represents the attribute (cosine squared). The HCPC used the first five dimensions representing 63% of the variance of the dataset; as this is considerably larger than the first two dimensions, the Figure 2c visually plots all attributes that differ between a cluster at *p*<.05.

The three clusters identified primarily differed by attributes associated with ripeness and texture. Cluster 3 is the smallest cluster (*n*=7) and has traits most related to unripe fruit—for example, vegetal/green and grassy flavors and astringent aftertaste. Cluster 2 is the next largest cluster (*n*=21) and is primarily classified by its negative textural attributes (e.g., hardness, roughness, toughness) and lack of strong flavors. Cluster 1 is the largest cluster (*n*=26) and is primarily classified by characteristics most related to ripe fruit—for example, “stone fruit/peach,” “floral/citrus,” and “toffee” flavors.

### 3.2 Durbin test separation on hedonic data from CT1

The ranking data from CT1 were analyzed using the Durbin test with LSD separation; the results are summarized in Table 2. The top ten ranked samples were not significantly different (*p*<.05) from the highest ranked sample (‘Nishimura Wase’) while the bottom seven products were not significantly different from the lowest ranked sample (‘Ichikeijiko’). There are examples of all astringency types (astringent, nonastringent, and variant) in both the top ten and bottom seven cultivars.

**Table 2:** Average rank and Durbin test separation results for the November 2015 consumer study (Consumer Test 1 [CT1])

Cultivar	Astringency type	Harvest date	Source	Average rank	Groups with same letter are not different (*p*=.05)
Nishimura Wase	V	10/12/2015	C‐1	4.00	a
Yotsumizo	A	10/8/2015	R	3.96	ab
Lycopersicon	A	10/22/2015	R	3.92	ab
Fuyu	N	10/8/2015	C‐3	3.63	abc
Jiro	N	9/22/2015	R	3.54	abcd
Maekawa Jiro	N	9/29/2015	R	3.54	abcd
Chocolate	V	10/20/2015	C‐1	3.50	abcd
Izu	N	9/10/2015	R	3.46	abcd
Izu	N	10/21/2015	C‐1	3.38	abcd
Lampadina	V	10/27/2015	R	3.21	abcde
Ichidagaki	A	10/1/2015	R	3.17	bcde
Syouro	V	10/22/2015	R	3.04	cdef
Fujiwaragosho	V	9/10/2015	R	2.96	cdefg
Chienting	V	9/24/2015	R	2.92	cdefg
Great Wall	A	9/24/2015	R	2.88	cdefg
Muraya	V	9/15/2015	R	2.83	cdefg
Fuyu	N	10/1/2015	R	2.79	defgh
Yeddo	N	10/8/2015	R	2.79	defgh
[unnamed]	A	10/6/2015	R	2.46	efghi
Emon	V	9/29/2015	R	2.42	efghi
F‐444	N	10/6/2015	R	2.42	efghi
Sangokuichi	V	9/17/2015	R	2.29	fghi
Mishirasu	A	9/15/2015	R	2.17	ghi
Korean	A	9/22/2015	R	2.00	hi
Ichikeijiko	A	9/17/2015	R	1.75	i

Higher value for Average Rank corresponds to more liking of the dried product (highest possible rank would be 5.00; lowest possible rank would be 1.00). Astringency Type: A, astringent; N, nonastringent; V, variant. Source: R, research plot (National Clonal Germplasm Repository, Davis, CA, USA); C‐X, commercial source X.

### 3.3 Combining trained panel and consumer data

#### 3.3.1 Establishing predictions using data from trained panel and CT1

**Figure 3:**
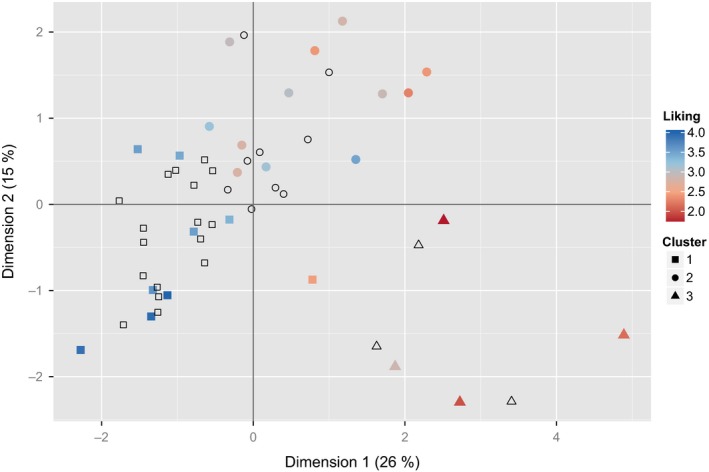
Multi factor analysis [MFA] biplot from trained panel data with overlay of consumer hedonic ranking data. Blue indicates a greater degree of liking; red indicates a lower degree of liking. Data points with no fill color represent samples that were not evaluated by the consumer group in Consumer Test 1

An analysis of variance along with Tukey's honest significant difference (HSD) was conducted to compare the sensory clusters’ mean likings. All clusters were different (HSD, *p*<.05), clusters and their mean liking are summarized in Supplemental Table S4. In addition, the consumer mean ranking from CT1 has been color coded on the MFA biplot in Figure 3. The mean ranking of the samples was most strongly correlated with the first dimension of the MFA (Spearman's Rho=−0.86, *p*<.0001).

Due to the strong correlation between hedonic ranking and the sensory MFA, a PLS regression model was created to relate consumer liking to the dried persimmons’ sensory profile. The PLS model has a coefficient of multiple determination of 0.7266, indicating consumer's mean hedonic ranking is well correlated with the sensory properties as determined by a trained panel. Supplemental Figure S2 presents the coefficients for the regression model from largest and smallest to better guide what attributes may influence liking. In brief ‐ the attributes “floral/citrus”, “squash”, “stone fruit/peach”, “sweet”, “minty”, “moistness”, “flavor intensity”, “spicy”, “toffee”, and “earthy” were positively correlated with high liking ranking. The attributes “woody”, “grassy”, “nutty”, “skin toughness”, “astringent”, “chlorine”, “vegetal/green”, “banana”, “fibrousness”, “sour”, “hardness”, “bitter”, “crispness”, “apple”, “chewiness”, “melon”, “vinegar”, “roughness”, “honey”, and “cucumber” were negatively correlated with liking ranking. Both the sensory cluster and the hedonic model are in agreement for what traits appear to be preferred by the consumer group; sensory characteristics associated with ripe fruits were preferred.

**Figure 4:**
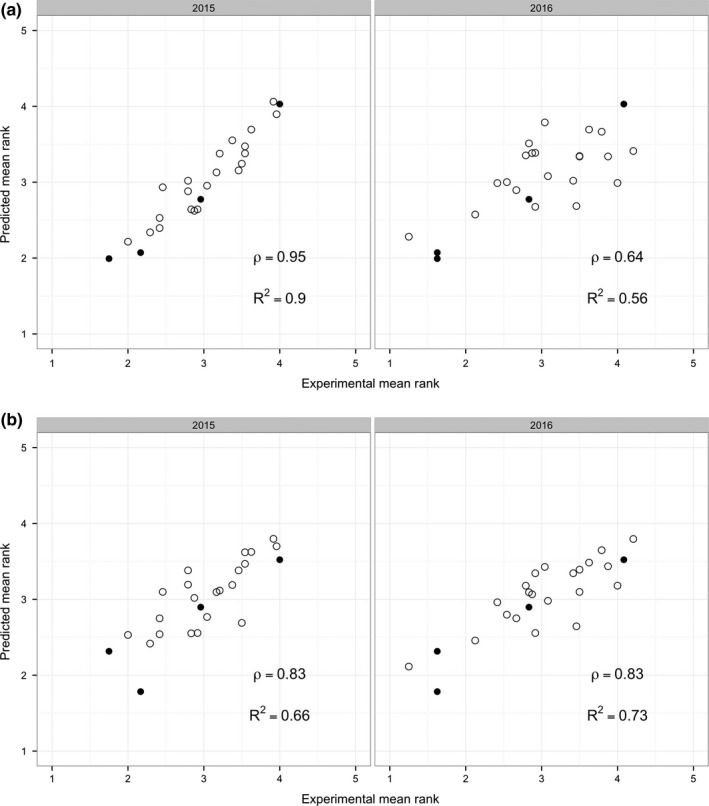
Predicted mean rankings vs. measured mean rankings of the samples for the full partial least squares regression (PLSR) model with all trained panel attributes included (a) and the sparse PLSR model, which excluded specific flavor attributes (b). The four samples which were common to both years are represented with filled data points

It is possible to use the combined characterization and hedonic data presented in Figures 3 and 4 to predict which persimmon cultivars would yield a dried chip‐style product that would be preferred by consumers, even if a particular cultivar was not represented in the set of 25 samples presented in CT1. In Figure 3, there are 17 samples in Cluster 1 (the best‐preferred cluster) that were not evaluated by the consumer group in CT1 (depicted by unfilled square data points). While all of these represented potentially preferred dried products, some were from the second or third harvests of cultivars whose first and/or second harvests yielded dried samples in Clusters 2 and 3. Put another way, a persimmon cultivar may yield a preferred dried product, but only from later in the harvest season. If a dried product were made from fruit harvested commercial ripe, but early in the season, that dried product would not be preferred by consumers. Thus, the more useful subset of the 17 consumer‐untested samples in Cluster 1 is comprised of cultivars whose trained panel MFA score placed them into Cluster 1 *at the first harvest*. There are 10 cultivars in this subset; they are listed below and highlighted on the MFA biplot in Supplemental Figure S3. In alphabetical order (no ranking information implied), the 10 cultivars that were not tested by consumers in Consumer Test 1 [CT1] but whose trained panel multi‐factor analysis [MFA] score placed them into Cluster 1 at the first harvest are ‘Akoumanzaki’, ‘Fennio’, ‘Fuyu Imoto’, ‘Fuyu Jiro’, ‘Giant Fuyu’, ‘Gofu’, ‘Hachiya’, ‘Maru’, ‘Suruga’, and ‘Tishihtzu’.

It should be noted that all of the commercial samples examined in this study fell into Cluster 1 (regardless of whether they were tasted during CT1). This indicates that persimmon cultivars currently on the market are either known to be or predicted to be a good starting material for production of a dried chip‐style product. ‘Fuyu’ persimmons from source C‐3 were in Cluster 1 and preferred in dried form by the consumer group. It is encouraging to see the closely‐related cultivars ‘Fuyu Imoto’, ‘Fuyu Jiro’, and ‘Giant Fuyu’ appear in the list. In the same pattern, ‘Jiro’ and ‘Maekawa Jiro’ chips from the research source were preferred by the CT1 consumer group, and the closely‐related cultivar ‘Fuyu Jiro’ from a commercial source appears in the list. So, there is some consistency in the cultivar groups that appear in Cluster 1.

#### 3.3.2 Confirming predictions using data from CT2

While the sample set was limited (due to harvest timing) for CT1 in November 2015, the entire sample set was available for CT2 in November 2016. The set of 25 samples for the latter test included 16 cultivars that had not been evaluated in the former test. Of these, nine cultivars had been confirmed to be preferred by consumers (the “a” group at top of Table 2), and 10 cultivars had been predicted to be preferred by consumers. The Durbin test separation results for CT2 are given in Table 3, along with the mean ratings. (Recall that both ranking and rating data were gathered in CT2, while only ranking data were gathered in CT1.) In Table 3, the 10 predicted‐to‐be‐preferred‐at‐first‐harvest cultivars are emphasized in bold font, and the high‐ (‘Nishimura Wase’), medium‐ (‘Fujiwaragosho’, first harvest), and low‐preference (‘Ichikeijiko’, first harvest) “anchor” samples are underlined.

**Table 3:** Average rank and Durbin test separation results for the November 2016 consumer study (Consumer Test 2 [CT2])

Cultivar	Astringency type	Average rank	Groups with same letter are not different (*p*=.05) [based on ranking]	Average rating
Mishirasu (3rd)	A	4.21	a	11.23
Nishimura Wase	V	4.08	ab	10.74
**Hachiya**	A	4.00	ab	9.55
Fuyu [Source C‐2]	N	3.88	ab	11.22
**Tishihtzu**	A	3.79	abc	9.73
**Fuyu Imoto**	N	3.63	abcd	10.46
Tamkam	N	3.50	abcde	10.30
**Gofu**	V	3.50	abcde	9.79
**Suruga**	N	3.46	bcde	10.26
**Akoumanzaki**	V	3.42	bcde	8.83
Okugosho	V	3.08	cdef	9.04
**Fennio**	A	3.04	def	9.04
Vainiglia	V	2.92	def	7.83
**Giant Fuyu**	N	2.92	def	8.54
**Fuyu Jiro**	N	2.88	ef	8.55
Fujiwaragosho (1st)	V	2.83	efg	7.92
Fujiwaragosho (2nd)	V	2.83	efg	8.57
**Maru**	N	2.79	efg	7.83
Mandarino	V	2.67	fg	7.09
Moro	V	2.54	fg	7.29
Hanagosho	N	2.42	fg	6.63
Ichikeijiko (2nd)	A	2.13	gh	6.54
Mishirashu (1st)	A	1.63	hi	4.26
Ichikeijiko (1st)	A	1.63	hi	4.17
Mishirashu (2nd)	A	1.25	i	2.70

Higher value for Average Rank corresponds to more liking of the dried product (highest possible rank would be 5.00; lowest possible rank would be 1.00). Higher value for Average Rating corresponds to more liking of the dried product (highest possible rating would be 15.00; lowest possible rating would be 0.00). Tukey's least significant difference results (lower case lettering) are based on ranking data. The predicted‐to‐be‐preferred‐at‐first‐harvest cultivars (based on CT1 data) are given in bold font. The “anchor” samples (based on CT1 data) are underlined. Cultivars for which multiple harvests were tested are indicated with the “1st,” “2nd,” or “3rd” modifier. Astringency Type: A, astringent; N, nonastringent; V, variant.

In CT2, all 10 of the predicted‐to‐be‐preferred‐at‐first‐harvest cultivars from CT1 had a mean rating above 7.5 (possible range of 1–15), indicating that these cultivars were indeed preferred and liked by consumers. However, some cultivars in this set fared better than others. ‘Hachiya’, ‘Fuyu Imoto’, ‘Gofu’, and ‘Tishihtzu’ were all in the group of not significantly different (*p*>.05) top eight samples for CT2. In contrast, ‘Akoumanzaki’, ‘Fennio’, ‘Fuyu Jiro’, ‘Giant Fuyu’, ‘Maru’, and ‘Suruga’ fell more toward the middle of the set. In addition, ‘Tamkam’, which had not been predicted to be preferred at first harvest from the results of CT1, was in the top eight in the rankings of CT2 and had the fifth highest mean liking rating. Thus, the original PLSR model (including all the sensory attributes evaluated by the trained panel) over‐predicted the performance of 6 cultivars and missed 1 cultivar.

In a more general way, the performance of the PLSR model can be assessed for all 25 samples presented to the consumers in each year. Figure 4a depicts the predicted mean rankings versus the measured mean rankings of the samples for the PLSR model with all trained panel attributes included. Four samples (three high‐, medium‐, and low‐preference “anchors” plus ‘Mishirashu’ first harvest) were common to both years; these are represented with filled data points. The remaining 21 samples were unique to each year.

With a Spearman's ρ of 0.95, the original PLSR model predicts the performance of samples from CT1 (from which the model was constructed) very well, but it does not fare as well in predicting the CT2 rankings (Spearman's ρ=0.64). However, when the 21 specific flavor attributes are removed from the PLSR model, the predictive ability for CT1 decreases somewhat (Spearman's ρ=0.83), while the predictive ability for CT2 increases dramatically (Spearman's ρ=0.83). This is shown in Figure 4b. Our hypothesis for why the exclusion of the specific flavor attributes has the observed positive effect on the predictive power of the PLSR model is that the flavor characteristics of the earlier samples are different enough from the later samples that these attributes do not predict consumer preference as well. The coefficients for the more sparse model are depicted in Supplemental Figure S4. In brief, the attributes of “astringency,” “crispness,” “skin toughness,” “fibrousness,” “hardness,” “bitterness,” and “sourness” are seen to be strong negative drivers of preference, while “moistness,” (overall) “flavor intensity,” and “sweetness” positively drive preference.

Using this refined PLSR model, the initially‐identified‐from‐CT1‐and‐MFA/HCPC list of 19 persimmon cultivars (‘Akoumanzaki’, ‘Chocolate’, ‘Fennio’, ‘Fuyu’, ‘Fuyu Imoto’, ‘Fuyu Jiro’, ‘Giant Fuyu’, ‘Gofu’, ‘Hachiya’, ‘Izu’, ‘Jiro’, ‘Lampadina’, ‘Lycopersicon’, ‘Maekawa Jiro’, ‘Maru’, ‘Nishimura Wase’, ‘Suruga’, ‘Tishihtzu’, and ‘Yotsumizo’) plus the newly‐identified‐from‐CT2 cultivar (‘Tamkam’) could be further refined. Using the sparse PLSR model, the predicted rankings for the following six cultivars were all >3.5: ‘Fuyu’, ‘Lycopersicon’, ‘Maekawa Jiro’, ‘Nishimura Wase’, ‘Tishihtzu’, and ‘Yotsumizo’. (Note: The third harvest of ‘Mishirasu’ also had a predicted ranking >3.5, but this still does not result in our recommending it for drying and is explained in Section 3.4). These six cultivars are the best targets for growers to focus on for persimmon production for a hot‐air dried product.

From a practical persimmon‐growing perspective, this list is encouraging. Two of the cultivars in this list (‘Fuyu’ and ‘Nishimura Wase’) came from commercial sources; this indicates that the persimmon trees needed to produce a preferred dried product are already available for purchase and propagation. The cultivars that are not yet commercially popular but are recommended by this study—‘Lycopersicon’, ‘Maekawa Jiro’, ‘Tishihtzu’, and ‘Yotsumizo’—present an opportunity for exploration of new (to the USA market) persimmon cultivars that can be propagated specifically for production of fruit destined for hot‐air drying.

### 3.4 Trajectory of cultivars over multiple harvests

**Figure 5:**
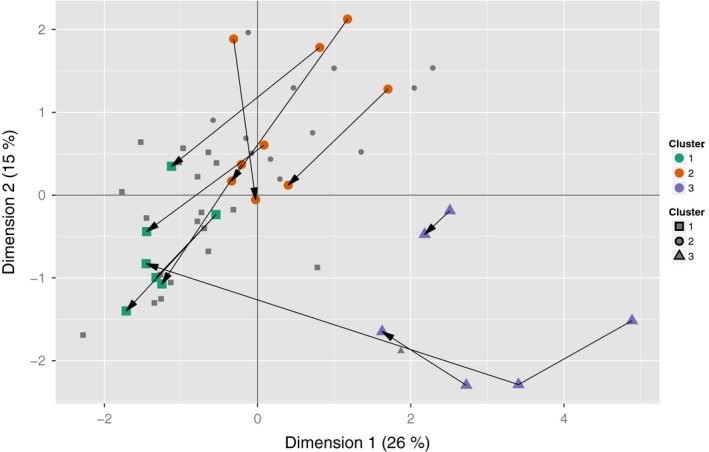
Trajectories of samples from multiple harvests depicted on the multi factor analysis (MFA) biplot. Large, colored points represent cultivars for which there were multiple harvests; small, grey points represent cultivars that were only harvested once during the study

In this study, some persimmon cultivars were harvested 2 or 3 times throughout the season. Figure 5 depicts the MFA biplot with all samples still shown at the same coordinates as in Figures 2a and 3 but with the cultivars that had multiple harvests depicted with larger, colored symbols and connected with arrows. For samples that had two harvests, the first and second harvests are at the plain and pointed end of the arrow, respectively. For samples that had three harvests, the first two harvests are connected by a plain line, and the second and third harvests are connected with an arrow.

From Figure 5, it is observed that the general trajectory of the samples was to move toward Cluster 1 (or deeper into Cluster 1) as the harvest season progressed. This suggests that consumers will generally prefer dried, chip‐style persimmon products from fruit harvested later in the season. This hypothesis was supported by data from the cultivar ‘Mishirasu’ during CT2. This cultivar had three harvests, of which only the first was used in CT1. In the MFA, ‘Mishirasu’ shows a distinct trajectory of staying in Cluster 3 for the first two harvests and then ending in Cluster 1 (preferred cluster) at the third harvest. Indeed, from the results of CT2 shown in Table 3, the mean rankings for ‘Mishirasu’ first and second harvest are at the very bottom of the list, while the mean ranking for the third harvest is at the very top. Despite the resulting conclusion to only use later‐harvest fruit for drying, this practice must be weighed against the possible decreased ease of slicing as the fruit get riper and softer. In this study, some of the latest‐harvest samples were nearly impossible to slice (even with a commercial meat slicer) because they were too soft. Thus, we recommend that persimmon growers use cultivars that yield consumer‐preferred dried products as soon as the fruit are commercial ripe (and still firm).

It should be noted that not all cultivars followed the pattern of clearly increasing in preference as the harvest season progressed. For the low‐ and medium‐preference anchors (‘Ichikeijiko’ and ‘Fujiwaragosho’, respectively), consumer preferences for the two harvests were not statistically different. It is possible that a third harvest of these cultivars would have yielded a more highly‐preferred product, but the issue of over‐ripeness hindering slicability would again be a concern.

The harvest‐timing effect is likely the cause of the conspicuously lower‐than‐expected ranking of ‘Fuyu’ fruit from Source R; see Table 2, where this sample had an average ranking of 2.79. This was a mediocre ranking, especially in comparison to that of the same cultivar from Source C‐3 (ranking=3.63, statistically tied with the top‐ranking sample in CT1). Although both ‘Fuyu’ samples—and all other samples in the study—were harvested when the fruit were commercial ripe, these two particular samples were clearly at different maturity levels at harvest, as evidenced by the greater amount of green skin color of the fruit from Source R. This is shown in the top two panels of Supplemental Figure S5. Indeed, the second harvest of ‘Fuyu’ from Source R, shown in the bottom panel of the figure, has much more even orange color. Although this sample was not part of the set tested in CT2, it is one of the samples projected to be in Cluster 1 in the MFA biplot (Figure 5), versus the Source R first harvest sample, which was in Cluster 2. So, ‘Fuyu’ remains a recommended cultivar for hot‐air drying, with the stipulation that—beyond commercial maturity—even orange skin color should be a prerequisite for ‘Fuyu’ fruit selected for this process.

## 4 CONCLUSIONS

The astringency type (astringent, nonastringent, variant) did not appear to inherently predict whether the dried chips made from a given persimmon cultivar would be preferred by consumers, since examples of all astringency types could be found throughout the ranking lists in both years. Thus, this attribute should not be used to screen persimmon cultivars for their suitability for hot‐air drying. Regarding harvest timing, the general trajectory of the samples over the harvest season was into (or further into) the most‐preferred cluster. However, we recommend using cultivars that are harvest‐date‐independent in their liking.

Comparison of the full and sparse PLSR models indicates that flavor is likely secondary to taste and texture in dried persimmon chips. Based on the sparse model, the six persimmon cultivars most suited for hot‐air drying (for fruit harvested commercial ripe at any time during the season) are the following: ‘Fuyu’, ‘Lycopersicon’, ‘Maekawa Jiro’, ‘Nishimura Wase’, ‘Tishihtzu’, and ‘Yotsumizo’. This list includes cultivars that are already established in the U.S. market as well as cultivars that have not yet seen widespread commercial propagation.

## CONFLICT OF INTEREST

Mention of trade names or commercial products in this article is solely for providing specific information and does not imply recommendation or endorsement by the US Department of Agriculture. USDA is an equal opportunity employer. The authors have no conflicts of interest to declare.

## ENDNOTE

1 In the horticulture literature, the “variant” category of persimmon cultivars contains two sub‐categories: Pollination‐Variant Astringent (PVA) and Pollination‐Variant Nonastringent (PVNA). In both sub‐categories, the flesh around the seeds changes color from orange to brown (Yonemori, Sugiura, & Yamada, 2000). For the purposes of this study, both PVA and PVNA cultivars are categorized as “variant”.
