# Supplementary figures and images for: Synthesis of descriptive sensory attributes and hedonic rankings of dried persimmon (Diospyros kaki sp.)
Source: Food Sci Nutr. 2017 Nov 9;6(1):124–36. doi: 10.1002/fsn3.537 (PMC5778222; doi:10.1002/fsn3.537)

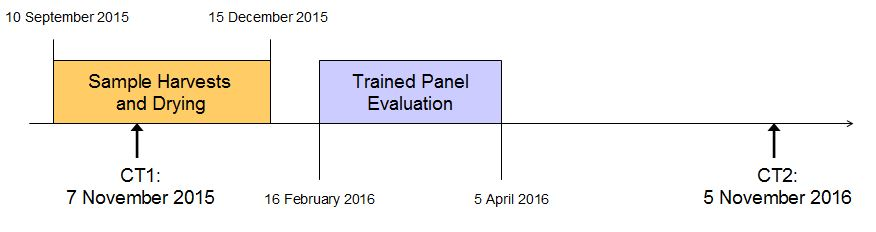

Supplement: Supplementary file 1 [file FSN3-6-124-s001.tiff]

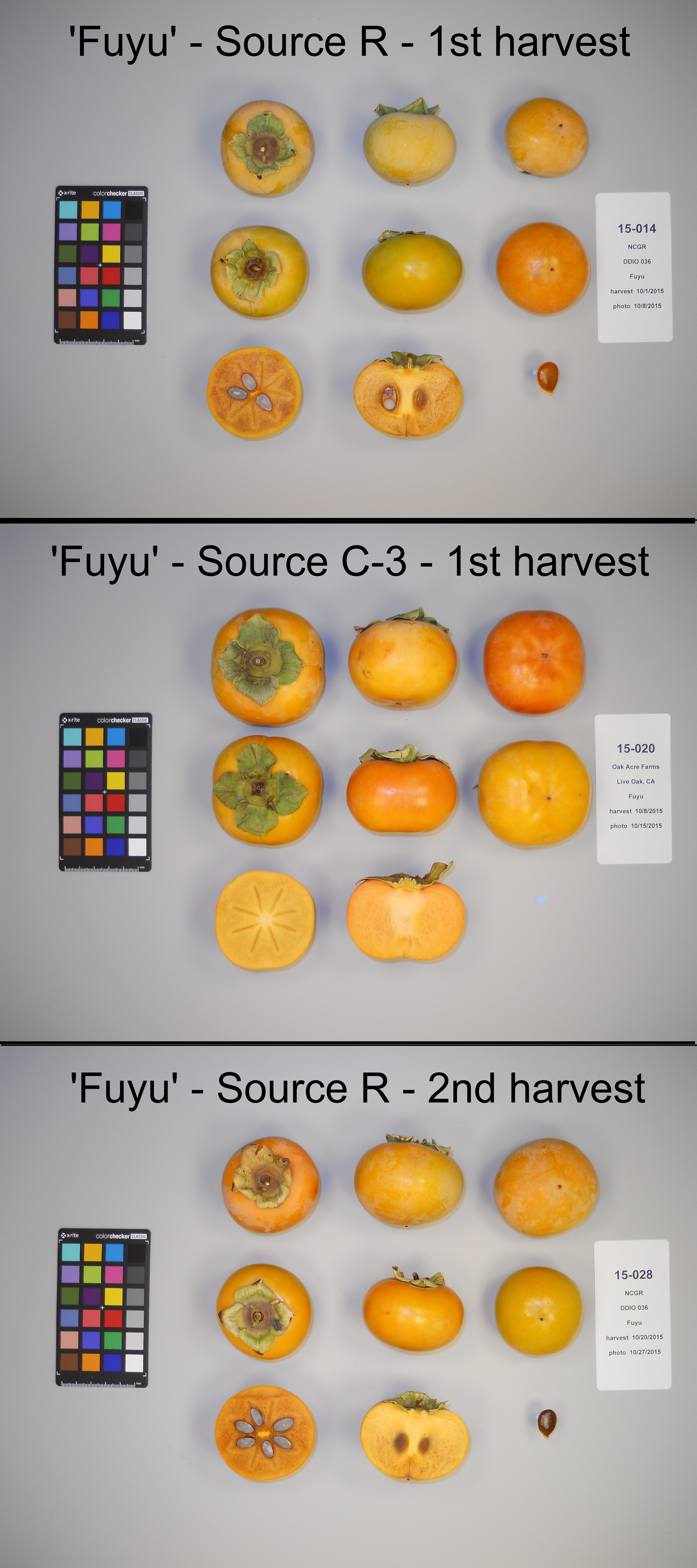

Supplement: Supplementary file 5 [file FSN3-6-124-s005.tiff]
